# Pain, fatigue, anxiety and depression in older home‐dwelling people with cancer

**DOI:** 10.1002/nop2.406

**Published:** 2019-11-05

**Authors:** Elena Solvik, Siri Ytrehus, Inger Utne, Ellen Karine Grov

**Affiliations:** ^1^ Department of Emergency Reception Ostfold Hospital Trust, Kalnes Grålum Norway; ^2^ Faculty of Health Western Norway University of Applied Sciences Bergen Norway; ^3^ Faculty of Health Sciences Institute of Nursing and Health Promotion Oslo Metropolitan University Oslo Norway

**Keywords:** cancer, elderly, home care nursing, nurses, nursing, older people, symptoms

## Abstract

**Aim:**

Globally, cancer incidence counts for more than 14 million cases and the number increases with age. The aim of this study was to investigate the occurrence of pain, fatigue, anxiety and depression in association with demographic and clinical factors.

**Design:**

A cross‐sectional descriptive design was used.

**Methods:**

We performed descriptive statistics to analyse the questionnaires completed from 174 older home‐dwelling people with cancer.

**Results:**

The people with cancer reported low occurrence of pain, fatigue, anxiety and depression. We found strong correlation between anxiety and depression. Women reported significantly higher scores of anxiety and depression than men. A higher pain score was associated with higher scores of fatigue and anxiety.

**Conclusions:**

Home care personnel meeting older home‐dwelling people with cancer should emphasize these people’ mental health problems and be aware that pain, fatigue and anxiety may occur at the same time.

## 1 INTRODUCTION

Cancer is a common disease with more than 14 million cases worldwide annually (Cancer Research UK), and those over 60 years are particularly effected. It is estimated that 36% of all men and 29% of all women will be effected by the age of 75 (Cancer Registry of Norway Institute of Population Based Cancer Research, 2018). The most common symptoms among people with cancer include pain, anxiety, depression (Edrington et al., 2010) and fatigue (Hwang, Cho, & Yoo, 2016; Oh, Seo, Jeong, & Seo, 2012; So et al., 2009).

### 1.1 Background

The symptom combination of pain, fatigue, anxiety and depression puts a high burden on the people with cancer. The consequences can be reduced ability to cope with activities of daily living (Grov, Fossa, & Dahl, 2010, 2017), which may lead to the need for help and support while living at home. People with cancer often face remarkable pain problems with varying origins, such as the disease itself and its treatment (Chang, Arnold, & Savarese, 2017; Dawn, 2011). There are several studies pointing out that one of the most common symptoms in people with cancer is pain (Posternak et al., 2016; Walsh, Donnelly, & Rybicki, 2000) and fatigue (Peters, Goedendorp, Verhagen, Van Der Graaf, & Bleijenberg, 2014; Shao et al., 2016; Wang et al., 2014; Zeng et al., 2012). It is estimated that about 33% of all people with cancer have moderate and severe pain, and for these people, everyday activities and social interactions may be challenging. According to International Association of the Study of Pain (2012), pain is described as the destruction of tissue as well as both sensory and emotional discomfort. This definition emphasizes that pain is not only result of tissue injury but effects emotional aspects as well.

Cancer illness effects the diseased person both physically and mentally due to the treatment side effects and the stress connected to the diagnosis (Dawn, 2011; Farrell, Brearley, Pilling, & Molassiotis, 2013). Anxiety and depression in people with cancer can reduce the quality of life and may contribute to worse disease outcome as compared people not experiencing mental health problems (Utne, Miaskowski, Bjordal, Paul, & Rustoen, 2010). Mental illness can be associated with increased mortality in people with cancer (Chan, Wan Ahmad, Yusof, Ho, & Krupat, 2015). Anxiety and depression in people with cancer are more frequently seen among women than men and among those having impaired activity status (Hellstadius et al., 2016). Fatigue is hard to understand due to its expression where the feeling of tiredness might be present despite completed treatment (Peters et al., 2014; Shao et al., 2016; Wang et al., 2014; Zeng et al., 2012).

Recent health reforms in Norway emphasize that the inhabitants should live at home for as long as possible, even when treated for serious illness. The local health authorities have to take responsibility of delivering health services to older people with cancer living at home. However, after this healthcare reform we need to know more about symptoms and complaints among those receiving health services from the community. The aim of this study was therefore to examine the occurrence of pain, fatigue, anxiety and depression in association with demographic factors in a selection of older home‐dwelling people with cancer.

For this sample, the following research questions were posed: (a) What is the prevalence of pain, fatigue, anxiety and depression? (b) Is there a difference between pain, fatigue, anxiety and depression scores in male and female participants? (c) Is there a difference between pain, fatigue, anxiety and depression among the oldest and the youngest participant groups? (d) Is there a difference in pain, fatigue, anxiety and depression for those in a paired relationship versus those living alone? (e) To what degree does pain correlate with fatigue, anxiety and depression? (f) What is the association between pain and demographics, clinical variables, fatigue and depression?

## 2 METHODS

A cross‐sectional descriptive design was used. The sample consisted of 174 home‐dwelling Norwegian people (mean age: 77.4 years) who had received their first‐time cancer diagnosis. The questionnaire was completed during discharge from the hospital to the home, or during the week after arriving home. Inclusion criteria for the participants were as follows: (a) ≥65 years of age; (b) living at home; and (c) needing assistance from home care nursing. Clinical variables are as follows: cancer diagnosis (type of cancer); time since diagnosis (dichotomized into “<1 year” and “≥1 year”); ongoing treatment (receiving cancer treatment: “yes” or “no”); functional level (dichotomized into “normal”—normal activity and “limited”—limited activity/up more than half part of the day, needing assistance/only in bed, needing full assistance); BMI (dichotomized into “≥22” and “<22”); and comorbidity (other diseases: “yes” or “no”). Demographic variables are as follows: age (dichotomized from median age of the sample: “≤77 years” and **“**>77 years”); civil status (dichotomized into “paired”—have a cohabitant/married, having a partner and “non‐paired”—widow/widower/separated/divorced); education (dichotomized into “<13 years” and “≥13 years”); and network (someone to talk to ‐ family or friends: “yes” or “no”).

The questionnaire, the Norwegian version of the Edmonton Symptom Assessment System (ESASr), is a psychometric tested instrument on symptom burden including pain, tiredness/fatigue, anxiety and depression (Bergh, Aass, Haugen, Kaasa, & Hjermstad, 2012; Bruera, Kuehn, Miller, Selmser, & Macmillan, 1991). The instrument has a scoring range from 0 (no complaints)–10 (worst complaints) and the assessments were self‐reported, as recommended by gold standard in research (Polit & Beck, 2017). Cross‐culturally the concept “tiredness” in the ESAS‐r is explained as “lack of energy” corresponding to the word “fatigue” (http://www.palliative.org/NewPC/-pdfs/ESAS-r.pdf). However, translation of instruments is challenging both linguistically and culturally (Utne et al., 2017), particularly when languages have no exact word for the phenomenon.

By means of the SPSS, version 24, we performed descriptive statistics such as frequencies, comparison between groups (*t* tests for continuous variables and Chi‐square for categorical variables) and linear regression analysis to determine possible associations between pain, fatigue, anxiety, depression and the demographic and clinical variables. Correlations between examined symptom levels were measured by Spearman's rho. Statistically significant levels were set at *p* ˂ .05 and all tests were two‐tailed.

### 2.1 Ethics

Research Ethics Committee approval was obtained by the Regional Committees for Medical and Health Research Ethics (REK) (REK: #1524). The patient protection advocate at each health institution and the chief medical officer in each municipality approved the study. The study adhered to the ethical research principles of the Declaration of Helsinki (World Medical Association, 2018). Data were kept under lock and key and will be stored at the Norwegian Social Science Data Services (NSD) after completing the study. The participants gave informed written consent. They were informed both verbally and in writing about their right to withdraw from the study.

## 3 RESULTS

People with cancer (*N* = 174) with a mean age of 77.4 years participated. Table 1 shows the comparison of demographic and clinical characteristics of the total and when divided by sex. There was a significant difference between men and women in terms of civil status, ongoing treatment, anxiety and depression. There were more single women (55% vs. 35%; *p* = .01) and more women who underwent treatment (52% vs. 35%; *p* = .04) than men. Average anxiety (2.34 vs. 1.28; *p* = .00) and depression (2.55 vs. 1.55; *p* = .01) scores were significantly higher in women than in men. Standard deviation was higher than average scores of symptoms pain, fatigue, anxiety and depression, which means there is a large spread in the study's sample. However, when comparing the groups, a non‐parametric test (Mann–Whitney *U* test) showed the same pattern as the parametric test and we therefore kept the *t* test for this presentation.

**Table 1:** Elderly people with cancer in Norway: sociodemographic and clinical characteristics of the total sample and by sex (*N* = 174)

	Total 172 (100%)	Male 71 (41%)	Female 101 (59%)	*p*‐value
Age, mean (*SD*)	77.4 (7.1)	77.8 (6.8)	77.0 (7.3)	.51
Age groups (%)				
≤77 years	88 (52)	33 (48)	55 (56)	.35
>77 years	80 (48)	36 (52)	44 (44)	
Civil status, *N* (%)				
Paired (married/cohabiting)	91 (53)	46 (65)	45 (45)	**.01**
Non‐paired (single/divorced/widow)	81 (47)	25 (35)	56 (55)	
Education, *N* (%)				
<13 years	129 (77)	49 (70)	80 (82)	.10
≥13 years	39 (23)	21 (30)	18 (18)	
Region Norway, *N* (%)				
West	45 (26)	17 (24)	28 (28)	.60
East	129 (74)	54 (76)	73 (72)	
Cancer diagnosis, *N* (%)				
Breast	12 (7)	0 (0.0)	12 (12)	**.00**
Prostate	20 (12)	20 (20)	0 (0)	**.00**
Lymphoma	10 (6)	4 (6)	6 (6)	1.00
Lung	14 (8)	5 (7)	9 (9)	.78
Colon	15 (9)	7 (10)	8 (8)	.79
Brain	3 (2)	1 (1)	2 (2)	1.00
Rectal	9 (5)	2 (3)	7 (7)	.31
Bladder	5 (3)	4 (6)	1 (1)	.16
Ovarian	12 (17)	0 (0)	12 (12)	**.00**
Other	21 (12)	12 (17)	9 (9)	.16
Network (someone to talk to), *N* (%)				
Yes	165 (97)	68 (97)	97 (97)	1.00
No	5 (3)	2 (3)	3 (3)	
Time since diagnosis, *N* (%)				
<1 year, *N* (%)	109 (64)	46 (66)	63 (63)	.75
≥1 year, *N* (%)	61 (36)	24 (34)	37 (37)	
Ongoing treatment, *N* (%)				
Yes	77 (45)	25 (35)	52 (52)	**.04**
No	95 (55)	46 (65)	49 (48)	
Functional level ECOG, *N* (%)				
Normal	37 (22)	14 (21)	23 (23)	.71
Limited/poor	130 (78)	54 (79)	76 (77)	
BMI, *N* (%)				
≥22	107 (63)	49 (70)	58 (59)	.15
<22	62 (37)	21 (30)	41 (41)	
Comorbidity (other diseases), *N* (%)				
Yes	89 (53)	30 (43)	59 (60)	**.04**
No	79 (47)	39 (57)	40 (40)	
Symptoms, mean (*SD*)				
Pain	2.41 (2.35)	2.31 (2.10)	2.45 (2.50)	.71
Fatigue	4.08 (2.58)	4.04 (2.46)	4.12 (2.68)	.84
Depression	2.12 (2.52)	1.55 (2.20)	2.55 (2.66)	**.01**
Anxiety	1.89 (2.43)	1.28 (1.95)	2.34 (2.64)	**.00**

Note

*p* < .05 is in bold.

Table 2 shows several significant but weak relationships, and the strongest relationship was between anxiety and depression. We therefore included only the anxiety variable in further regression analysis due to the high correlation between anxiety and depression and to avoid multi‐collinearity. Including both variables in regression analysis could disturb the results as a kind of tautology.

**Table 2:** Correlation matrix: pain, fatigue, anxiety and depression (*N* = 174)

	Pain	Fatigue	Anxiety	Depression
Pain	–	0.31**	0.34**	0.31**
Fatigue	0.31**	–	0.37**	0.38**
Anxiety	0.34**	0.37**	–	0.76**
Depression	0.31**	0.38**	0.76**	–

** *p* < .01.

**Figure 1:**
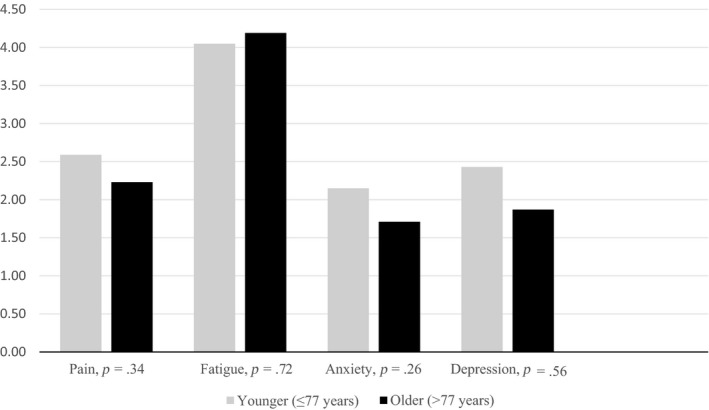
Pain, fatigue, anxiety and depression in younger and older people with cancer (*N* = 174)

**Figure 2:**
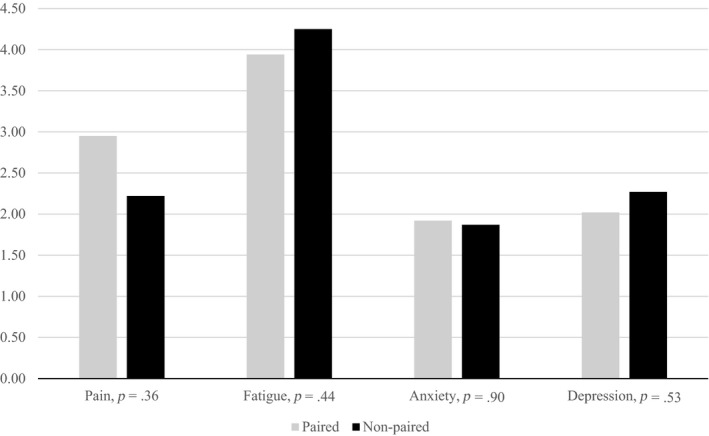
Pain, anxiety, depression and civil status in paired and non‐paired people with cancer

Table 3 shows that higher pain score was significantly associated with higher levels of fatigue and anxiety. Bivariate regression analysis revealed *p* = .05 for fatigue. However, we decided to include fatigue in further multivariate regression analysis because this *p*‐value was on the threshold of becoming significant. The difference in examined symptoms between the older and the younger patient groups was not statistically significant and similar findings revealed in the comparison of people in paired and non‐paired relationship (Figures 1 and 2).

**Table 3:** Bivariate and multivariate linear regression analyses with pain as dependent variable

Independent variables	Bivariate analyses			Multivariate analyses		
	Stand. *β*	*t*	*p*	Stand. *β*	*t*	*p*
Fatigue	0.17	1.94	**.05**	0.25	3.34	**.001**
Anxiety	0.20	2.30	**.02**	0.27	3.62	**<.001**
Age (dichotomized by median value)	−0.09	−1.16	.25			
Gender	0.02	0.26	.79			
Civil status (paired/not paired)	−0.06	−0.73	.47			
Comorbidity (other illnesses, yes/no)	0.13	1.68	.10			
BMI (≥22/<22)	0.07	0.94	.35			
Functional level (normal/poor, limited)	0.15	1.88	.06			
Ongoing treatment (yes/no)	0.07	0.93	.35			
Time since diagnosis (<1 year/≥1 year)	−0.11	−1.36	.18			
Education (<13 years/≥13 years)	0.04	0.46	.65			

Note

*p* < .05 is in bold.

## 4 DISCUSSION

The prevalence for anxiety, depression and pain in this sample of older home‐dwelling people with cancer showed low mean values (1.9, 2.1 and 2.4, respectively) which indicate a low symptom burden. This finding indicates that for this sample substantial treatment in addition to what is already offered is not needed since the cut‐off value for pain is ≥4, indicating moderate and severe symptom burden and for anxiety and depression ≥2 (Vignaroli et al., 2006). The low symptom burden reported by the participants may be due to appropriate treatment. Of note, the variation among the participants in reporting symptoms highlights the importance to assess these symptoms to find out who needs substantial treatment. For the symptom fatigue, the mean score was above four, which indicates that relevant interventions might be needed. While we have used the item “tiredness” from ESAS‐r as equivalent with “fatigue”, more information is needed weather the people with cancer in this sample need interventions for fatigue or not.

### 4.1 Symptoms in male and female participants

The women in our study reported significantly higher levels of two of the four symptoms, namely anxiety and depression. Several studies that examined people with cancer have documented that women experience higher emotional distress than men (Aass, Fosså, Dahl, & Moe, 1997; Linden, Vodermaier, Mackenzie, & Greig, 2012; Mystakidou et al., 2006). Additionally, women reported higher levels of fatigue (Husson et al., 2015; Kim, Yen, & Rabow, 2016; Pashos et al., 2013), anxiety (Aass et al., 1997) and depression (Cardoso, Graca, Klut, Trancas, & Papoila, 2016; Linden et al., 2012; Mystakidou et al., 2006).

Despite the large samples in latter mentioned studies, the comparison with our findings has some limitations. For example, Aass et al. (1997) divided people with cancer into different age groups, just one of these is a relevant age group (≥65 years) for our study. In addition, the women in the study by Pashos et al. (2013) were significantly older than men, which could explain the higher fatigue scores reported by the women. In the study of Husson et al. (2015), where they used the fatigue measure “The Fatigue Assessment Scale”, the instrument and the sample of only cancer survivors may account for their findings. In Cardoso et al.'s (2016) study, they evaluated symptoms in 270 people with cancer with a mean age of 59 years the same day as the first chemotherapy session started. Depression was reported by 24% and anxiety by 30% of the participants and being a woman was an independent risk factor for both symptoms. These findings could be explained by the fact that some of the participants previously had received a psychologist follow‐up. Additionally, the interviews were conducted the same day as the first chemotherapy sessions started which could create stress. Linden et al. (2012) had a sample of people with cancer with a mean age of 59 years, not matching the mean age in our study sample.

According to Landi et al. (2005), more women experienced daily pain compared with men. Although not including people with cancer, we consider this study relevant for this discussion due to the sample of older home‐dwelling people. However, sample differences can to a certain degree explain dissimilarity in the results. Cancer‐related pain might be more prominent than non‐cancer pain, independent of sex. Other studies examining people with cancer found that women reported more pain compared with men. In a study by Kim et al. (2012), the sample's age groups were not equally distributed (those <65 years accounted for 70%), possibly biasing the results. The Korean origin of the study makes it difficult to compare the results due to differences in culture and in health services. Kim et al. (2016) had a low sample size and remarkable large attrition rate (127 of 205 participated). In Nahin's (2015) US population study, English‐speaking women reported more pain than men, while both males and females from other ethnic groups reported almost similar pain levels. These findings emphasize the limited ability to compare studies from different countries or continents.

### 4.2 Symptoms in oldest and youngest participant groups

In this sample of older home‐dwelling people with cancer, there was no significant difference in anxiety, depression, pain and fatigue between the oldest and youngest participants. Our findings are consistent with a previous report. Based on analyses of symptom cluster fatigue, anxiety and depression in older and younger people with cancer, researchers concluded that this symptom combination did not depend on age (Agasi‐Idenburg, Thong, Punt, Stuiver, & Aaronson, 2017). However, several studies show increased risk of mental disorders among older people (Buigues et al., 2015; Drageset, Eide, & Ranhoff, 2013; Landi et al., 2005; Mystakidou et al., 2006). For example, many studies showed that higher age is associated with lower anxiety levels (Austin, Wiley, McEvoy, & Archer, 2011; Linden et al., 2012; Wochna Loerzel, 2015). Other studies showed that higher age is a risk factor for depression (Cardoso et al., 2016; Fujisawa et al., 2016). Most likely, this is not depression caused by age but impaired physical health due to comorbidity and other age‐related health problems.

Other studies show that the oldest people reported less pain than the youngest. For example Morita et al. (2014) assumed that lower pain incidence in the oldest participants may be due to reduced sensitivity to symptoms or to over‐ or under‐reported symptoms. Older people may underestimate pain or perceiving pain as an inevitable part of disease or ageing.

Husson et al. (2015) presented lower fatigue levels in the oldest participant group (>65 years) than in the youngest group (≤65 years). Morita et al. (2014) found higher fatigue levels in the oldest people as compared with the youngest, but the average age of this sample was significantly lower than in our study. Husson et al. (2015) examined long‐term cancer survivors, which does not correspond with our sample. It is reasonable to expect that symptoms change due to the time since diagnosis and with the number of treatment lines (Peters et al., 2014). The inconsistent findings of differences between the oldest and the youngest participants in these four symptoms highlight the importance of assessing demographics and settings in the different studies.

### 4.3 Pain, fatigue, anxiety, depression and civil status

It is interesting to note that we did not found any differences in examined symptoms between those who were in paired‐relationships and those who lived alone. A contrasting result was found in a study by Husson et al. (2015), where people living alone reported significantly higher fatigue scores than those with a partner. A possible explanation might be that those who live alone have no one at home to share everyday activities; they might become more tired compared with those with partners who most likely share the daily chores. Another plausible explanation for the divergent findings could be related to the sample, where only 32% of the participants were above the age of 70. A third explanation might be the assessment tool (Fatigue Assessment Scale) which does not correspond with the single item from ESAS‐r used in our study.

In studies among people with breast cancer, support from one's surroundings turns out to have a positive effect on psychological well‐being (Salonen et al., 2013; Yoo et al., 2014). We also assume that people, who have someone nearby most of the time, receive more support because of the partner's availability. Therefore, our results are surprising. A possible explanation might be presence of the home care nurses compensating support from others.

### 4.4 Combination and connection between pain, fatigue, anxiety and depression

Relatively low levels of anxiety and depression among the participants in this study are consistent with the findings of another study examining older people with cancer (Harrison et al., 2011). As in our study, a study by Agasi‐Idenburg et al. (2017) revealed a strong significant correlation between depression and anxiety. The correlation matrix that includes pain, fatigue, depression and anxiety showed the correlation between anxiety and depression to be the strongest.

Several studies confirm association between pain, anxiety, depression (Landi et al., 2005; Utne et al., 2010) and fatigue (Paiva et al., 2012). Some presented the relationship between pain and anxiety (Mystakidou et al., 2006) and some between pain and depression (Bair et al., 2004; Kim, Malone, & Barsevick, 2014; Laird, Boyd, Colvin, & Fallon, 2009; Landi et al., 2005). The study of Paiva et al. (2012) used an ESAS‐scale for symptom measurement, the same as in our study. In the study by Landi et al. (2005), the pain was registered by using health personnel's observation evaluation (proxy rating) which may give an under‐ or overestimation of the person's symptoms (Blomqvist & Hallberg, 1999).

Even though several studies confirm different associations among these four symptoms, there is no clear evidence of a causal relationship between pain, fatigue, anxiety and depression. According to a population study, people who are suffering from continuous pain are in greater risk of experiencing poor health and disability than those who are in less severe pain (Nahin, 2015). Another study shows strong connection between pain and fatigue in people with cancer undergoing treatment. Those who had both pain and fatigue tended to experience higher degree of depression (Kim, Shaffer, Carver, & Cannady, 2014).

It is possible that well‐adjusted pain treatment may reduce fatigue, anxiety and depression (Kim et al., 2013) and individual follow‐up programmes may have a positive effect on symptoms like fatigue, anxiety (Paiva et al., 2012), pain and depression (Shi et al., 2015). These explanations are pointing out the connection between the person's physical and mental states. Other studies show the link between physical and mental health and argues that untreated pain can result in increased anxiety and depression levels in people with cancer (Mystakidou et al., 2006). For example, one study found a strong correlation between people with cancer's depression symptoms and impaired physical activity (Kim & Yi, 2015). This connection can be explained by the possible side effect of cancer treatment. Fatigue may result in impaired physical activity, leading to the loss of normal daily activities and to mental distress. This understanding may be linked to findings in two other studies. People with cancer with lower activity levels are at greater risk of developing depression (Grov, Fossa, & Dahl, 2010). Older people's lack of physical activity and impaired social roles may predict anxiety and fatigue can lead to depression (Aass et al., 1997). A more advanced study methodology is needed to confirm the hypothesis that pain, fatigue, anxiety and depression are connected and to understand the possible causal relationship between these symptoms. However, the theoretical assumption that mental and biological processes are connected (Kandel, 1998) seems to be supported in this study. It is therefore worth to shed light on the nursing perspective claiming the person's condition and surroundings to incorporate physical, physiological and mental aspects (Schaller, Larsson, Lindblad, & Liedberg, 2015).

Based on our findings and the results of previous studies, we assume that to be a woman may result in increased mental distress. Therefore, women facing cancer should be given attention by health care personnel in the community.

### 4.5 Limitations of the study

The use of a single item to measure symptoms is a limitation of this study. However, in a review of the measurement of psychological distress in palliative care, the authors reported that assessment measures like Edmonton Symptom Assessment Scale (ESAS), play an important role in clinical research (Kelly, McClement, & Chochinov, 2006). In addition, Hotopf, Chidgey, Addington‐Hall, and Ly (2002) noted that a single‐item questionnaire has obvious advantages in palliative care populations.

We had no possibility of cross‐checking the self‐rated symptom burden with their healthrecords. More female participants reported living alone and undergoing treatment than male participants. This could explain why some symptoms were more prominent in women than in men. At recruitment, only 77 participants were receiving treatment; this could result in low average scores of pain, anxiety and depression.

A large variety of pain, fatigue, anxiety and depression assessment tools have been used to measure symptoms in previous studies. Further, differences in demographic and clinical variables of the samples (e.g. differing age groups), different study aims and methods create a challenge when comparing results.

## 5 CONCLUSIONS

Physical and mental processes in people with cancer are linked, and this connection have been confirmed by several studies. The main results from our study present a strong association between anxiety and depression as well an association of pain with fatigue and anxiety. Home care personnel meeting older home‐dwelling people with cancer should emphasize the person's mental health problems. Focus on the complexity of the symptom burden and awareness of pain, fatigue and anxiety symptoms occurring at the same time is important.

More studies are needed to investigate causal relationship between these symptoms and sex differences. We recommend a larger sample of older home‐dwelling people with cancer than used in this study with particular focus on cluster analyses.

## CONFLICT OF INTEREST

The authors declare that they have no competing interests.

## AUTHOR CONTRIBUTIONS

Ellen Karine Grov (EKG) and Siri Ytrehus (SY): Conception of the protocol for the project and designing of the survey. Elena Solvik (ES) and EKG: Conduction of the analyses and designing of the tables and figures. ES: Writing of the manuscript with guidance from the other authors (EKG, SY and Inger Utne (IU)). All authors (ES, SY, IU and EKG) have read and approved the final manuscript.

## Data Availability

The data file supporting the conclusions of this article will be available in the Norwegian Centre for Research Data (NSD) (https://www.nsd.uib.no/nsd/english/index.html), after completing the study. Data can be shared with readers on request to the corresponding author.
